# Characterization of Diaphanous-related formin FMNL2 in human tissues

**DOI:** 10.1186/1471-2121-11-55

**Published:** 2010-07-15

**Authors:** Maria Gardberg, Kati Talvinen, Katja Kaipio, Kristiina Iljin, Caroline Kampf, Mathias Uhlen, Olli Carpén

**Affiliations:** 1 Department of Pathology, University of Turku and Turku University Central Hospital, Turku, Finland; 2 Medical Biotechnology, VTT Technical Research Centre and University of Turku, Finland; 3 Human Protein Atlas Project, Uppsala University, Uppsala, Sweden

## Abstract

**Background:**

Diaphanous-related formins govern actin-based processes involved in many cellular functions, such as cell movement and invasion. Possible connections to developmental processes and cellular changes associated with malignant phenotype make them interesting study targets. In spite of this, very little is known of the tissue distribution and cellular location of any mammalian formin. Here we have carried out a comprehensive analysis of the formin family member formin -like 2 (FMNL2) in human tissues.

**Results:**

An FMNL2 antibody was raised and characterized. The affinity-purified FMNL2 antibody was validated by Western blotting, Northern blotting, a peptide competition assay and siRNA experiments. Bioinformatics-based mRNA profiling indicated that FMNL2 is widely expressed in human tissues. The highest mRNA levels were seen in central and peripheral nervous systems. Immunohistochemical analysis of 26 different human tissues showed that FMNL2 is widely expressed, in agreement with the mRNA profile. The widest expression was detected in the central nervous system, since both neurons and glial cells expressed FMNL2. Strong expression was also seen in many epithelia. However, the expression in different cell types was not ubiquitous. Many mesenchymal cell types showed weak immunoreactivity and cells lacking expression were seen in many tissues. The subcellular location of FMNL2 was cytoplasmic, and in some tissues a strong perinuclear dot was detected. In cultured cells FMNL2 showed mostly a cytoplasmic localization with perinuclear accumulation consistent with the Golgi apparatus. Furthermore, FMNL2 co-localized with F-actin to the tips of cellular protrusions in WM164 human melanoma cells. This finding is in line with FMNL2's proposed function in the formation of actin filaments in cellular protrusions, during amoeboid cellular migration.

**Conclusion:**

FMNL2 is expressed in multiple human tissues, not only in the central nervous system. The expression is especially strong in gastrointestinal and mammary epithelia, lymphatic tissues, placenta, and in the reproductive tract. In cultured melanoma cells, FMNL2 co-localizes with F-actin dots at the tips of cellular protrusions.

## Background

The formin family consists of large multidomain proteins that control cytoskeletal organization [1]. Formins are conserved in all eukaryotes, where they govern complex cellular processes such as cell shape and motility, migration and cytokinesis. The human genome contains 15 formin genes that are subdivided in Diaphanous-related formins (DRFs) and non-DRFs. The protein family is defined by the formin homology 2 domain (FH2), capable of polymerization straight actin filaments. FH2 attaches to the actin filament during elongation, protecting it from capping proteins. Almost all formins contain a proline-rich formin homology 1 domain (FH1) N-terminal to FH2. FH1 enhances actin nucleation by recruiting profilin-bound actin monomers for FH2 actions.

Formin-like 2 (FMNL2), a member of the DRFs, contains a GTPase-binding domain and autoregulatory domains, and is proposed to function as a downstream effector of Rho family guanosine triphosphatases (GTPases) [2]. Rho GTPases influence cell morphology by activating effectors in different cellular compartments. As most formins, the mouse FMNL2 orthologue is known to polymerize actin filaments [3].

Furthermore, DRFs are tied to transcriptional regulation, mediating the activation of serum response factor (SRF). SRF activation in turn, leads to transcription of several cytoskeleton-associated genes [4]. Recently, a microdeletion including the FMNL2 gene was associated with precocious puberty, short stature and severe mental retardation [5].

As actin regulation is a fundamental process, inherited or acquired defects in this complex activity can result in different diseases. These include developmental disorders, muscle disorders, various neoplasias, immune deficiencies, diseases of the nervous system and kidney diseases. As key regulators of cytoskeletal dynamics, formins are interesting not only from a biological but also from a disease perspective. Despite intensive studies, the distinct functions of different formins remain unresolved. FMNL2 mRNA is expressed in many normal tissues and cancers [6]. Surprisingly, the presence of FMNL2 protein has not systematically been studied by immunohistochemistry in any species, possibly due to the lack of antibodies suitable for such analysis. The aim of this study was to determine the expression profile of FMNL2 in normal human tissues, using an antibody designed for immunohistochemistry. The expression pattern can be used as a reference in further analyses of FMNL2 biological functions and disease associations.

## Methods

### mRNA expression analysis

The GeneSapiens database was utilized to study the FMNL2 mRNA expression across all human normal tissues [7]. All the samples included in this database have been analyzed on the Affymetrix platform and due to unique normalization and data quality verifications, gene expression profiles collected from different studies can be combined to generate an overview of the expression profile in human tissues.

### Tissue samples and immunohistochemistry

Tissue samples were collected for this study prospectively from surgical specimens sent to the Department of Pathology at the Turku University Central Hospital for diagnostic purposes. Informed consent was asked before surgery. The procedure was approved by the Hospital District Ethics Committee. Within one hour from surgical removal, macroscopically normal tissue areas were sampled. Central nervous tissue specimens were an exception, as autopsy material was collected. Tissues were fixed in formalin, dehydrated, paraffin-embedded and sectioned.

Immunostaining was performed using the streptavidine-peroxidase method, using a LabVision autostainer device. For antigen retrieval, the slides were treated with citrate buffer (pH 6.0). Endogenous peroxidase was blocked with 3% H_2_O_2_. After 30 min incubation in normal nonimmune serum at 37°C, the sections were incubated 60 min at 4°C in affinity-purified rabbit FMNL2 antibody (Atlas Antibodies, Stockholm, Sweden; 1:75 dilution). After washing with PBS, the sections were treated with biotin-conjugated secondary antibody, followed by streptavidine-peroxidase. Diaminobenzidine was used as a chromogene. Individual tissues and cell types were evaluated by scoring the staining intensity by grading from 0 (no staining) to + + + (strong staining).

### Northern hybridization of human cell lines

Total RNA was isolated from the cell line samples using Trizol Reagent (Invitrogen, Carlsbad, CA), according to manufacturer instructions. The concentration of RNA was determined spectrophotometrically and the quality was analyzed by agarose gel electrophoresis and ethidium bromide staining by evaluating the integrity of 28S and 18S ribosomal RNAs. Northern blotting, hybridization and the detection of the hybridized probe was performed according to DIG Application Manual for Filter Hybridization (Roche Applied Science, Mannheim, Germany) with some modifications. Briefly, 1.0 μg of total RNA was size-fractionated on a denaturing 1.3% agarose/formaldehyde gel and stained with ethidium bromide. RNA was blotted to a positively charged nylon membrane (Roche Applied Science) by capillary transfer. RNA was treated with 50 mM NaOH before transfer. The membrane was UV-crosslinked (UV Stratalinker™ 2400, Stratagene, La Jolla, CA) and equal quantity of transfered RNA samples was checked with ethidium bromide. After prehybridization for an hour in high SDS hybridization buffer [50% deionized formamide, 5 × SSC, 50 mM sodium phosphate pH 7, 7% SDS, 0.1% N-lauroylsarcosine, 2% Blocking Reagent (Roche Applied Science)] at 50°C, the membrane was hybridized in the same solution overnight at 50°C with a denaturated DIG-11-UTP labelled human FMNL2 RNA probe (DIG RNA Labelling Kit SP6/T7, Roche Applied Science). The sequence of the probe matched the antibody epitope (NCBI RNA RefSeq clone GenBank: NM_052905.3, bases 1544 - 1871). The final washing conditions were 0.5 × SSC, 0.1% SDS at 65°C for two times 15 min. The amount of hybridized probe was visualized with alkaline phosphatase labelled Anti-Digoxigenin Fab Fragments (Roche Applied Science) and chemiluminescent detection using CDP-*Star *substrate (Roche Applied Science).

### Western blotting of human cell lines

Cells from seven different human lines (MCF-7 = breast carcinoma, HaCaT = keratinocyte, A431 = squamous cell carcinoma, MKN-1 = gastric carcinoma, WM164 = melanoma, Jurkat = T-cell lymphoma, K562 = erythroleukemia) were harvested and lysed in ELB buffer (50 mM HEPES, pH 7.4, 150 mM NaCl, 5 mM EDTA), 1% Nonidet P-40 or in RIPA buffer supplemented with protease inhibitors. Samples were normalized for protein concentration and equal amounts of material in Laemmli sample buffer were subjected to SDS-PAGE, transferred to nitrocellulose filters and immunoblotted with FMNL2 antibody (1:2000, 60 min) in BSA/TBS/Tween 0,1%, followed by secondary antibody [HRP-conjugated swine anti-rabbit (Dako, Glostrup, Denmark)]. Bound proteins were detected by enhanced chemiluminescence. To verify the specificity of the FMNL2 antibody, its reactivity was blocked by 1 hour preincubation with a 100-fold molar excess of the GST-FMNL2 - fusion protein (residues 393-516), or as a control, with GST. The α-tubulin antibody, used as a control for protein loading, was from Invitrogen.

### FMNL2 small interfering (si) RNA

FMNL2 expression was silenced in MKN-1 and WM164 cells using *SMART*pool siRNA (Dharmacon Research, Boulder, CA). Non-targeting Pool siRNA (Dharmacon) was used as a control. Cells were transfected with Dharmafect 1 (Dharmacon) according to manufacturer's instructions. FMNL2 levels were examined in cell lysates 72 hours after transfection by immunoblotting.

### Immunocytochemical analysis

WM164, A431 and MKN-1 cells were cultured on coverslips and fixed with 4% paraformaldehyde for 15 min. The cells were permeabilized in cold acetone for 5 min. After 15 min in 1% BSA in PBS, the cells were incubated for one hour with the FMNL2 antibody (1:150), followed by a secondary antibody, Alexa 568-conjugated goat anti-rabbit (Molecular Probes, Eugene, OR). F-actin was visualized with Alexa 488-conjugated phalloidin (Molecular Probes). The mounting media contained DAPI for staining nuclei (Vector Laboratories, Burlingame, CA). In control slides the primary antibody was replaced with 1% BSA in PBS. The cells were imaged with a Zeiss LSM 510 Meta confocal microscope (Carl Zeiss, 63 × Plan Apochromat objective, Göttingen, Germany). Image J 1.42q (NIH, USA) software was used in picture analyses.

## Results

### Antibody validation

The FMNL2 rabbit anti-human polyclonal monospecific antibody was produced by the Swedish Human Proteome Resource (HPR), in a project with emphasis on generating antibodies for immunohistochemistry [8]. An epitope specific for FMNL2 (Uniprot Q96PY5, amino-acids 393-516) was chosen for recombinant protein production of Protein Epitope Signature Tags (PrESTs) and for immunization. Rabbit polyclonal antibodies were affinity purified and initially validated by ELISA-based PrEST-array of antibody specificity. In this assay, the antibody was highly specific.

**Figure 1:**
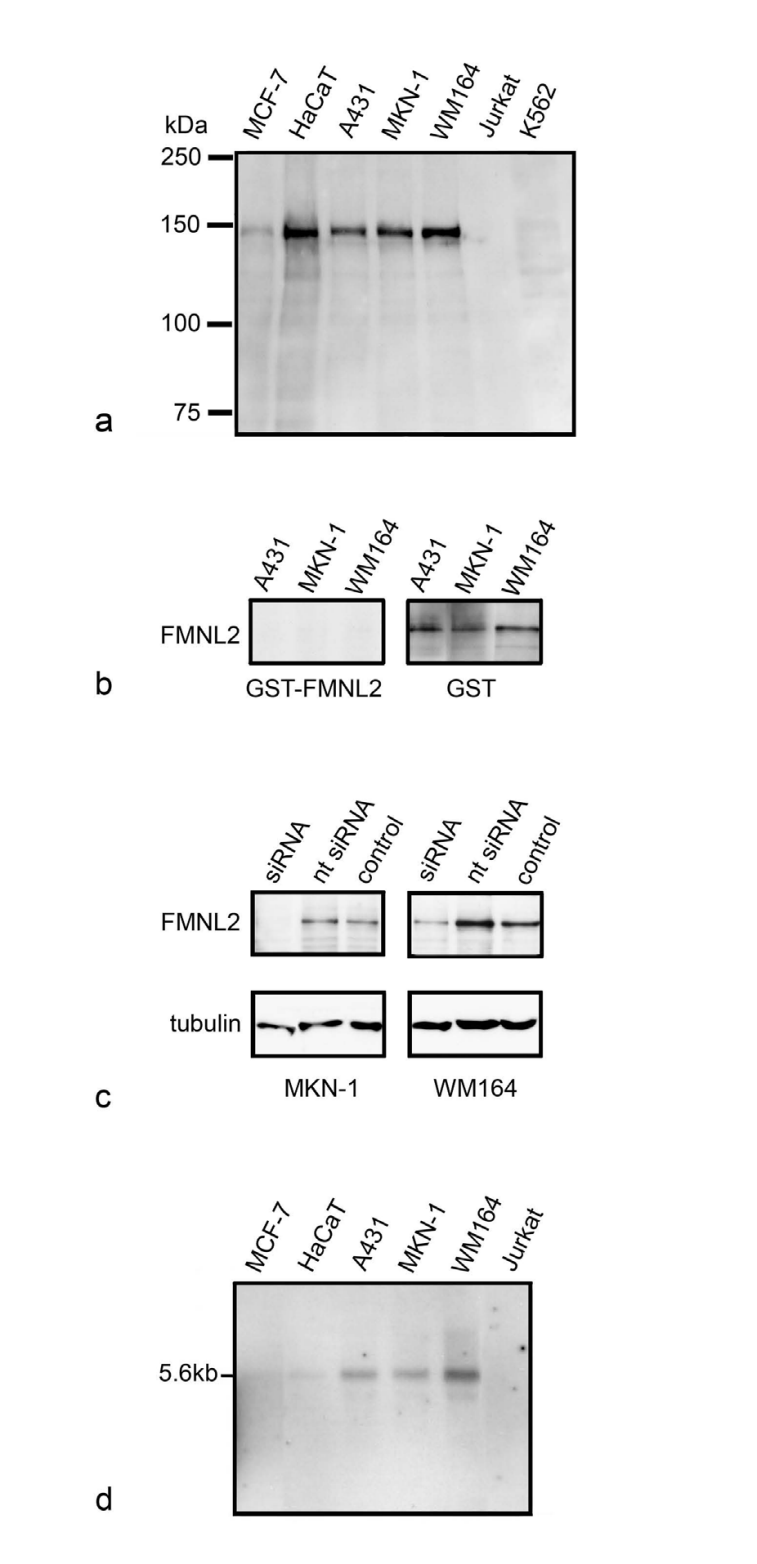
**Validation of FMNL2 antibody**. a) Western blot analysis of FMNL2 protein in seven human cell lines. A single band, of approximately 140 kDa, is detected in five cell lines. b) Western blot analysis with FMNL2 antibody competed either with GST-FMNL2 fusion protein or GST. Competition with GST-FMNL2 but not GST results in disappearance of the 140 kDa FMNL2 band. c) Western blot analysis of WM164 and MKN-1 cells transfected with FMNL2-specific siRNA, non-targeting (nt) siRNA or without siRNA (control). FMNL2 siRNA markedly reduces FMNL2 reactivity in both cell types. The α-tubulin blot confirms equal protein loading. d) Northern blot analysis of FMNL2 mRNA in six human cell lines. A 5.6 kb band is seen in all five cell lines, in which the Western blot analysis detects the protein band.

To further validate the FMNL2 antibody, Western and Northern blot analyses were carried out on several cell lines. Based on sequence information, the predicted molecular mass of FMNL2 is 124 kDa. The FMNL2 antibody detected a single band slightly higher than expected size (140 kDa) in the human cell lines MCF-7, HaCat, A431, MKN-1 and WM164. No band was detected in Jurkat and K562 cells (Figure 1a). The reactivity of the antibody was confirmed by a peptide competition assay. Preincubation of the antibody with GST-FMNL2 fusion protein resulted in loss of the 140 kDa band in all three tested cell lines, A431, MKN-1 and WM164, whereas preincubation with GST did not alter the reactivity (Figure 1b).

As a further test, WM164 and MKN-1 cells were transfected with FMNL2-specific siRNA or with control siRNA, and lysates were blotted with the FMNL2 antibody. FMNL2 knockdown resulted in markedly reduced or undetectable antibody reactivity, whereas control siRNA had no effect on antibody reactivity (Figure 1c).

The FMNL2 transcript in the cultured cells was analyzed by Northern blotting. The analysis revealed a major 5.6 kb transcript in all four cell lines, in which FMNL2 was detected by Western blotting. In A431, MKN-1 and WM164 cells an additional weaker and smaller band was seen. In Jurkat cells, no transcript was detected (Figure 1d).

### FMNL2 mRNA expression in human tissues

**Figure 2:**
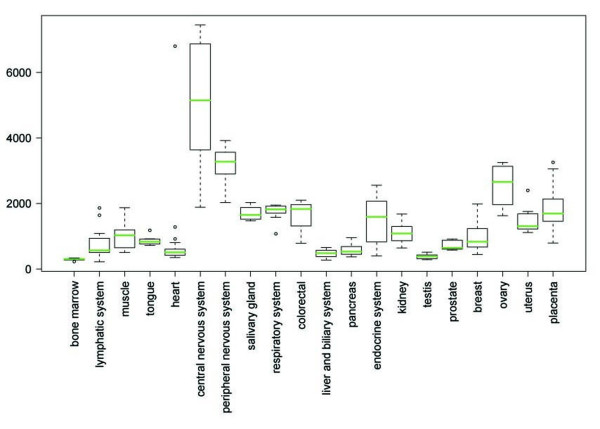
**FMNL2 mRNA expression profile in normal human tissues**. The normalized expression values (y-axis) of FMNL2 across normal tissues (x-axis) are presented as box-plots. The box extends from the first to the third quartile of the data and the median is indicated with green. The whiskers extend to the extreme values unless there are outliers. The data observations that lie more than 1.5 * interquartile range (IQR) lower than the first quartile, or 1.5 * IQR higher than the third quartile are considered as outliers and indicated separately. Highest expression levels are seen in central and peripheral nervous systems.

To gain an overall picture of FMNL2 mRNA expression in various human tissues, gene expression data from GenesSapiens database was utilized. FMNL2 mRNA expression profile across human tissues is presented in Figure 2. Overall, FMNL2 mRNA was widely expressed. The highest mRNA expression was seen in central and peripheral nervous systems. Relatively high expression was detected in ovary, placenta, colorectal tissues, respiratory and endocrine systems, and salivary gland. The lowest expression of FMNL2 was seen in bone marrow, testis, liver/biliary system, heart and pancreas [9].

### Immunohistochemical analysis of FMNL2 in human tissues

**Figure 3:**
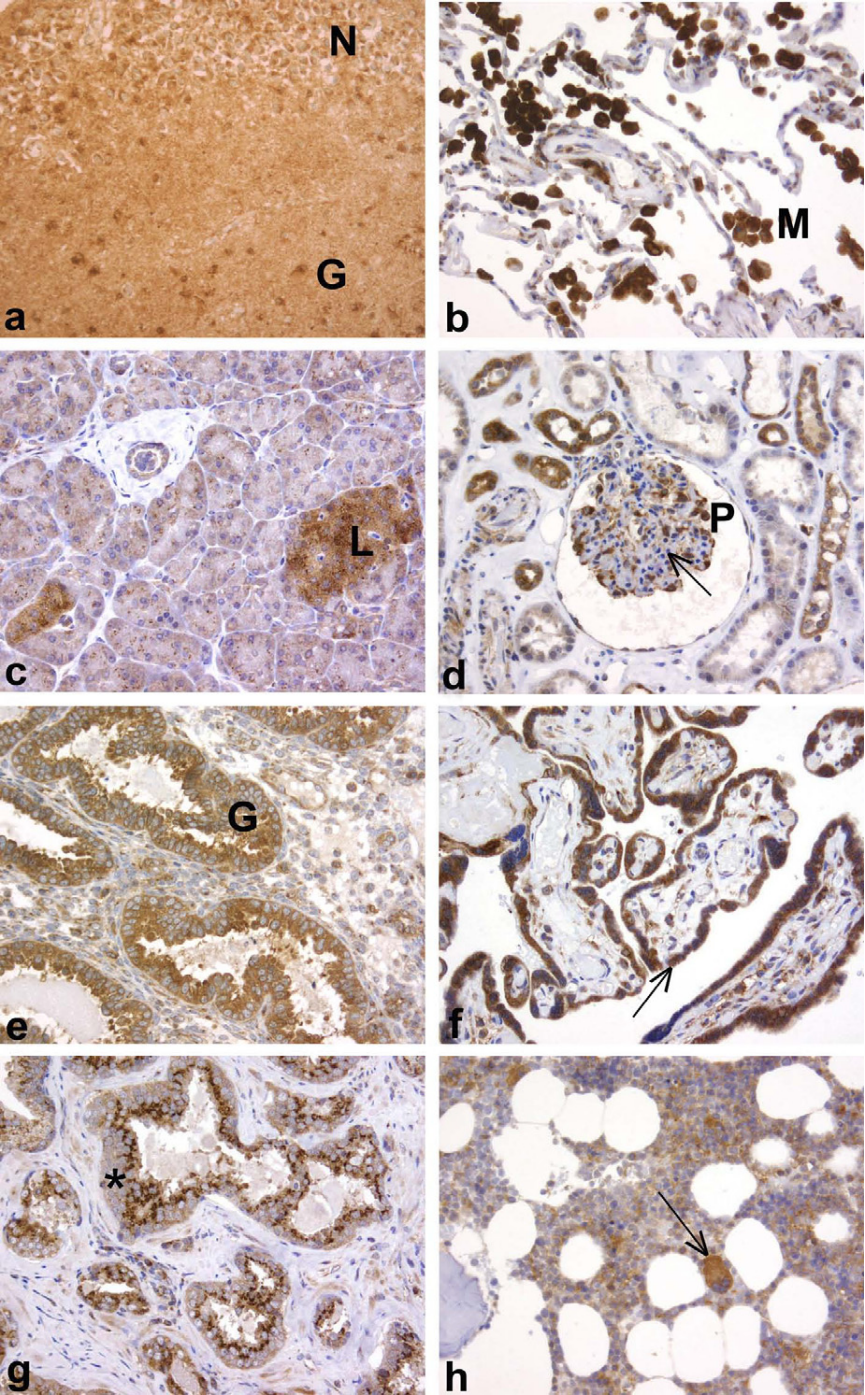
**Immunohistochemical analysis of FMNL2 in human tissues**. a) In the hippocampus, both neurons (N) and glial cells (G) express FMNL2 strongly. b) In the lung, FMNL2 expression is strong in macrophages (M), but weak in alveolar epithelium. c) The exocrine pancreas stains weakly and granularly for FMNL2. The neuroendocrine cells in the Langerhans islets (L) stain strongly. d) In the kidney, glomerular mesangial cells (arrow) do not expess FMNL2. In contrast, podocytes (P) and part of the tubules express FMNL2. e) In secretory endometrium, FMNL2 expression is strong in glandular epithelium (G), but weak in the stromal cells. f) In the placenta, trophoblasts (arrow) express FMNL2 strongly. g) In the prostate, epithelial cells stain intensely, with an apical perinuclear dot (asterisk). h) In the bone marrow, the strongest FMNL2 expression is seen in megakaryocytes (arrow). (**a - h **original magnification × 200)

Immunohistochemical analysis of 26 different human tissues was performed, and the results are summarized in Table 1. Examples of the staining results and cellular distribution of FMNL2 are shown in Figure 3. As an overall impression, most tissues and cell types expressed FMNL2 although the staining intensity varied. The subcellular location of FMNL2 was cytoplasmic and the distribution appeared to be uniform.

**Table 1:** Expression of FMNL2 in various tissues and cell types

Tissue	Cell type	Staining*	Comments
**Adrenal gland**	Cortical cells	+++	Partly granular

	Medullary cells	+++	Partly granular

**Bone marrow**	Erythroid cells	0	

	Myeloid cells	++	

	Megakaryocytes	+++	

**Breast**	Ductal epithelium	+++	

	Lobular epithelium	+++	

	Myoepithelium	++	

**Brain**	Neurons	+++	

	Glial cells	+++	

**Colon**	Epithelium	+++	

**Duodenum**	Surface epithelium	+++	Perinuclear apical dot

	Brunner glands	++	

	Stromal cells	+	

**Esophagus**	Squamous epithelium	+	Basal layer ++, surface layer 0

**Kidney**	Podocytes	+++	

	Mesangial cells	0	

	Tubular epithelium	++	Variable

**Liver**	Hepatocytes	+	

	Biliary duct epithelium	+	

**Lung**	Alveolar epithelium	+	

	Alveolar macrophages	+++	

**Lymph node**	Follicular cells	+++	

	Paracortical cells	+++	

**Ovary**	Granulosa cells	+++	

	Stromal cells	++	

**Pancreas**	Exocrine epithelium	+	Partly granular

	Ductal epithelium	+	

	Endocrine cells	+++	

**Parathyroid gland**	Chief cells	+++	

	Oxyphil cells	+	

**Parotid gland**	Acinar cells	++	Partly granular

	Adipocytes	0	

**Placenta**	Trophoblast	+++	

	Stromal cells	+	

	Endothelium	0	

**Prostate**	Epithelium	++	Perinuclear apical dot

	Smooth mucle cells	+	

	Peripheral nerve cells	++	

**Skin**	Keratinocytes	+	Basal layer ++, surface layer 0

**Small intestine**	Enterocytes	+++	

**Spleen**	White pulp cells	+++	

	Red pulp cells	+++	

**Stomach**	Glandular cells	+++	Partly granular apically Surface epithelium weak

	Stromal cells	+	

**Striated muscle**	Myocytes	+	

**Testis**	Spermatocytes	+++	

	Sertoli cells	+++	

	Leydig cells	+	

**Thyroid gland**	Epithelium	+++	

**Urinary bladder**	Urothelial cells	++	Weak in umbrella cells

**Uterus**	Smooth muscle cells	++	Perinuclear dot

	Secretory epithelium	+++	

	Secretory stromal cells	+	

*0 = no staining, + = weak staining, + + = moderate staining, + + + = strong staining

#### Central nervous system

In the brain, both nerve cells and glial cells expressed FMNL2 widely and abundantly (Figure 3a). In samples taken from 41 different areas of the brain, moderate to strong staining was seen in neurons, oligodendroglial cells and astrocytes alike.

#### Respiratory system

In the lung, expression was strong in alveolar macrophages, but weak in alveolar epithelium (Figure 3b). The respiratory epithelium in bronchi expressed FMNL2 strongly.

#### Gastrointestinal system

FMNL2 expression was strong in epithelia of the stomach, duodenum, jejunum, and colon. Moderate expression was seen in the glandular cells of parotid gland.

In the esophagus, pancreas and liver, staining was weak indicating low expression. However, the pancreatic neuroendocrine cells of Langerhans islets showed intense immunoreactivity (Figure 3c). While most cell types had uniform cytosplasmic staining, the secretory epithelia stained granularly and enterocytes had a particularly strong apical perinuclear dot. Ganglion cells, representing the peripheral nervous system, expressed FMNL2 abundantly.

#### Endocrine organs

Samples from thyroid gland, parathyroid gland and adrenal gland were studied. In all three locations, FMNL2 was strongly expressed. Expression was equally strong both in the cortex and medulla of the adrenal gland. Follicular epithelial cells of the thyroid gland and parathyroid chief cells also expressed FMNL2 strongly. Parathyroid oxyphil cells, in contrast, stained weakly.

#### Urinary system

Samples from kidney and urinary bladder were analyzed as examples of the urinary system. FMNL2 was present in both tissues. In the kidney, mesangial cells did not express FMNL2. Podocytes, on the other hand, expressed FMNL2 abundantly (Figure 3d). Tubular epithelial cells varied in intensity, most tubules stained moderately. In the urinary bladder, urothelial cells expressed FMNL2 moderately. Intensity was weaker in umbrella cells than in basal cells.

#### Female reproductive system

In the fertile ovary, FMNL2 was expressed in several cell types. Granulosa cells surrounding the maturing oocyte expressed FMNL2 strongly. The oocyte itself, as well as stromal cells, expressed FMNL2 moderately. In the uterus, myometrial smooth muscle cells expressed FMNL2 moderately. A perinuclear particularly strongly staining dot was noted. Secretory phase endometrial glands expressed FMNL2 strongly, stromal cells weakly (Figure 3e). In the non-lactating breast, FMNL2 was strongly expressed in both ductal and lobular epithelium. Myoepithelial cells stained moderately. In the full term placenta, both syncytiotrophoblast and trophoblast cells expressed FMNL2 strongly, whereas the staining of stromal cells was weak (Figure 3f).

#### Male reproductive system

FMNL2 immunoreactivity was detected both in testis and prostate. In testis, strong expression was seen both in spermatocytes and Sertoli cells. Leydig cells, in contrast, expressed FMNL2 weakly. In the prostate, epithelial cells expressed FMNL2 moderately, with an especially strong perinuclear apical dot (Figure 3g). Smooth muscle cells stained weakly. Moderate staining was seen in prostatic peripheral nerves.

#### Haematopoietic system

Samples from lymph node, spleen and bone marrow represented the hematopoietic system. In all these tissues, FMNL2 was detected. In both the lymph node and spleen, FMNL2 was widely and strongly expressed. In the bone marrow, erythroid cells did not express FMNL2. Megakaryocytes, on the other hand, expressed FMNL2 abundantly. Myeloid cells stained moderately (Figure 3h).

#### Skin

FMNL2 was present in epidermal keratinocytes and adnexal structures. In the epidermis, basal layers stained moderately. Expression gradually weakened toward the surface. Superficial cells did not express FMNL2.

#### Vasculature

In vessel walls, FMNL2 expression was weak or negative in endothelial cells. In muscular arteries, also smooth muscle cells stained weakly.

#### Skeletal muscle

In striated muscle, weak FMNL2 expression was detected. FMNL2 was uniformly distributed, without a striated pattern.

### Subcellular distribution of FMNL2 in WM164, A431 and MKN-1 cells

**Figure 4:**
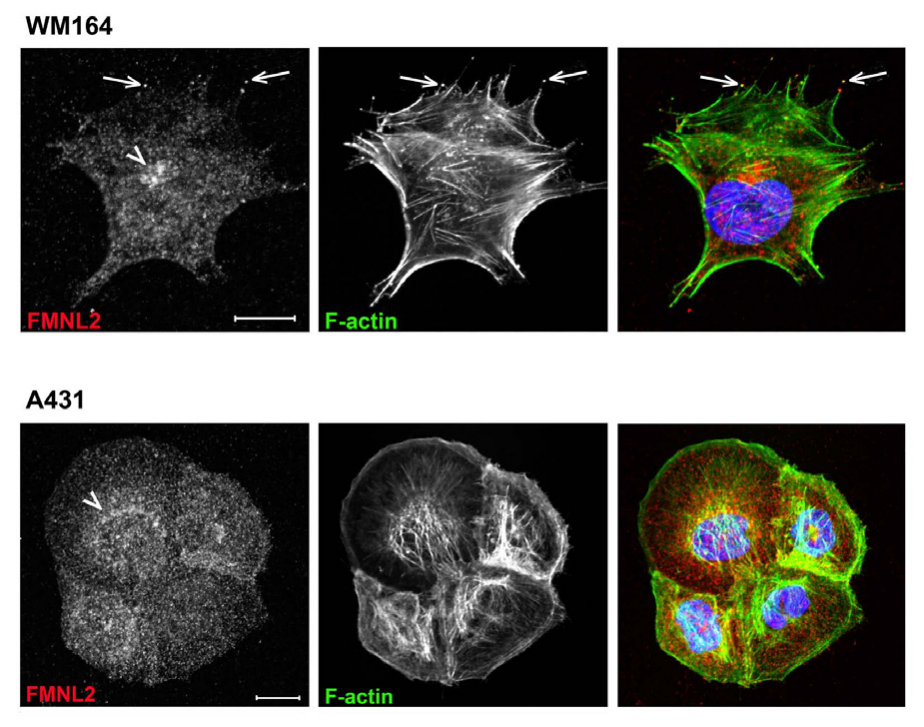
**Confocal microscopic analysis of FMNL2 in WM164 melanoma cells and A431 squamous cell carcinoma cells**. FMNL2 (left, red) was visualized with antibody, F-actin (middle, green) with phalloidin, the nucleus with DAPI (blue) and the cells analyzed with a confocal microsope. Top row: In WM164 cells, FMNL2 is located both diffusely in the cytoplasm and perinuclearly (blue) consistent with the location of the Golgi apparatus (arrowhead). WM164 cells have prominent protrusions with long actin filaments. At the tips of these protrusions, FMNL2 and F-actin are co-localized (arrows). Bottom row: A431 cells lack protrusions. FMNL2 is seen in the cytoplasm, faintly concentrated to the perinuclear location (arrowhead). No co-localization with F-actin is evident. Bar = 20 μm.

To study the subcellular location of FMNL2 in detail, WM164, A431 and MKN-1 cells were stained with FMNL2 antibody, phalloidin and DAPI. Confocal microscopic analysis revealed a primarily cytoplasmic distribution of FMNL2 in all three cell types. FMNL2 often showed perinuclear accumulation in WM164 and A431 cells, consistent with the Golgi apparatus (Figure 4, arrowheads). A similar staining pattern was occasionally observed in MKN-1 cells (not shown).

In MKN-1 and A431 cells, FMNL2 staining did not co-localize with F-actin, visualized by phalloidin staining. Instead, in WM164 cells a partial overlap was seen. In contrast to the other cell types WM164 cells contained protrusions with long actin filaments. Dots containing both FMNL2 and F-actin were observed at the tips of these protrusions in most of the analyzed cells (Figure 4, arrows).

## Discussion

The expression of different formin proteins in human tissues has not been systematically investigated. As the protein family has 15 members, the proteins are expected to possess individual and different functions and expression patterns, reflecting the demands for cytoskeletal regulation in various cell types. Here, we set out to investigate how widely FMNL2 is expressed by using immunohistochemistry. An antibody was raised for this purpose. After validation by Western and Northern blotting, as well as a peptide competition and siRNA experiments, we conclude that this antibody is specific and suitable for immunohistochemistry.

FMNL2 mRNA has been reported to be expressed in many normal tissues [6]. Using a large set of information on FMNL2 transcripts, we confirmed that FMNL2 mRNA indeed can be detected in virtually all tissues [7]. Highest expression was seen in the central nervous system, followed by the peripheral nervous system and several epithelial tissues. A consistent result was achieved by immunohistochemical analysis of 26 tissues. In the central nervous system, expression was wide, as strong expression was seen both in neurons and supporting glial cells. Strong expression was, however, not exclusive for nervous tissues. Moderate or strong expression was seen in most epithelia and lymphatic tissues. Especially gastric, intestinal, mammary and endocrine epithelia expressed FMNL2 strongly. Connective tissue components including fibroblasts, endothelium and adipose cells expressed little or no FMNL2. It will be interesting to study whether FMNL2 expression changes in invasive cancer, when epithelial cells lose their polarity and cell-to-cell contacts.

In the hematopoietic system, lymphocytes, myeloid precursors and macrophages displayed moderate to strong FMNL2 expression. Erythroid precursors, as well as mature red blood cells were negative. Consistent with this, no FMNL2 was detected by Western blotting in the K562 erythroleukemia cells. Such cell-type specific expression in cells differentiating from a common precursor may have clinical implications. Another DRF, Diaphanous 1 (mDia1), has been linked to bone marrow disease. mDia1 knockout mice develop age-dependent myeloproliferative defects mimicking human myeloproliferative and myelodysplastic syndromes [10]. FMNL2 could also be involved in diseases with defects in myelopoiesis. However, the phenotype of an FMNL2 knockout mouse has not been described.

One of the epithelia expressing FMNL2 was colon. The expression was relatively high both as analyzed by mRNA signal and immunohistochemistry. Our findings are distinct from the results reported in a study linking increased FMNL2 expression to metastatic activity in colorectal cancer. In that study, normal colorectal epithelium was reported to be devoid of FMNL2 or express it only weakly [11]. The difference may be due to several causes. The previously reported antibody may have a lower sensitivity than ours, resulting in positive immunoreactivity only in tissues with exceptionally high FMNL2 expression. Also, the antibodies may have different isoform-specifity. The antibody used for the colorectal cancer metastasis study was directed toward an antigen in FMNL2 isoform 1 that shares 91% identity with FMNL2 isoform 2. It is possible that the antibody has weaker or no affinity to FMNL2 isoform 2. In contrast, the antibody used in our study was raised towards a sequence common to both isoforms. The colorectal metastasis study illustrated the relative change of FMNL2 expression in normal colon and colorectal adenocarcinoma, especially metastatic disease. At this point, we conclude that normal colorectal mucosa does express FMNL2, but do not compare the level of expression to cancer. Thus, our results do not necessarily contradict the conclusions made. Our preliminary transcription analysis indicates that FMNL2 expression is up-regulated in several forms of cancer, and future studies will be directed to find the ones where this difference has biological and medical relevance.

Uncontrolled proliferation, combined with invasive and metastatic potential, is a hallmark of malignancy. Rho GTPases have been shown to mediate cytoskeletal changes essential for malignant cell behavior in vitro, and evidence of in vivo relevance is mounting. Rho GTPases activate effector molecules in different cellular locations. Effectors, in turn, mediate cytoskeletal changes [12]. Identifying Rho GTPase effectors is crucial for understanding actin remodeling in normal and malignant cells. FMNL2 contains a GTPase binding domain. Recently, FMNL2 was identified as a specific RhoC effector [13]. In the same study, RhoC and FMNL2 were, by using RNA interference, found to be essential for the invasive motility of rounded human melanoma cells. FMNL2 silencing did not affect the invasive capacity of breast carcinoma cells or fibrosarcoma cells, which display a more mesenchymal elongated morphology. In our study, confocal microscopy of WM164 melanoma cells revealed a co-localization of F-actin and FMNL2 at the tips of cell protrusions. A431 squamous cell carcinoma cells and MKN-1 gastric carcinoma cells had a different, unspiked morphology and in these cells FMNL2 and F-actin were not co-localized. These results are in agreement with the finding that FMNL2 has cell-specific functions and that it has a special role in protrusions of invading melanoma cells.

As Rho GTPase expression levels change in malignancies, changes in effectors are to be expected. Without doubt, several other formins will in the near future be identified as effectors specific Rho GTPases. Understanding the cascades behind malignant cell behavior in different malignancies can ultimately offer targets for novel therapies.

## Conclusions

With a novel FMNL2 antibody, we show that immunohistochemistry is a useful method of investigating FMNL2 expression in human tissues. In healthy tissues, FMNL2 is widely expressed. Highest expression is in the central nervous system. In melanoma cells, FMNL2 shows a distinct co-localization with actin dots in cellular protrusions. Studies on FMNL2 in cancer tissues are warranted.

## Competing interests

The authors declare that they have no competing interests.

## Authors' contributions

MG gathered samples, analyzed data and did the siRNA experiments, MG and OC designed the study, KT performed Northern blot experiments, KK performed the peptide competition experiment and confocal microscopy, KI collected bioinformatics data, CK validated and initially characterized the antibody, MU was project leader of the HPR project, MG and OC wrote the paper.

All authors read and approved the final manuscript.

## References

[B1] Faix J Grosse R Staying in shape with formins Dev Cell 2006 10 693 706 10.1016/j.devcel.2006.05.001 16740473

[B2] Higgs HN Formin proteins: a domain-based approach Trends Cell Biol 2005 30 342 353 10.1016/j.tibs.2005.04.014 15950879

[B3] Vaillant DC Copeland SJ Davis C Thurston SF Abdennur N Copeland JW Interaction of the N- and C-terminal autoregulatory domains of FRL2 does not inhibit FRL2 activity J Biol Chem 2008 283 33750 33762 10.1074/jbc.M803156200 18835814 PMC2662283

[B4] Young KG Copeland JW Formins in cell signaling Biochim Biophys Acta 2009 in press 10.1016/j.bbamcr.2008.09.017 18977250

[B5] Lybaek H Ørstavik KH Prescott T Hovland R Breilid H Stansberg C Steen VM Houge G An 8.9 Mb 19p13 duplication associated with precocious puberty and a sporadic 3.9 Mb 2q23.3q24.1 deletion containing NR4A2 in mentally retarded members of a family with an intrachromosomal 19p-into-19q between-arm insertion Eur J Hum Gen 2009 17 904 910 10.1038/ejhg.2008.261 PMC2986486 19156171

[B6] Katoh M Identification and characterization of human FMNL1, FMNL2 and FMNL3 genes in silico Int J Oncol 2003 22 1161 1168 12684686

[B7] The GeneSapiens Database http://www.genesapiens.com

[B8] Human Protein Atlas http://www.proteinatlas.org/

[B9] Kilpinen S Autio R Ojala K Iljin K Bucher E Sara H Pisto T Saarela M Skotheim RI Bjorkman M Mpindi JP Haapa-Paananen S Vainio P Edgren H Wolf M Astola J Nees M Hautaniemi S Kallioniemi O Systematic bioinformatic analysis of expression levels of 17,330 human genes across 9,783 samples from 175 types of healthy and pathological tissues Gen Biol 2008 9 R139 10.1186/gb-2008-9-9-r139 PMC2592717 18803840

[B10] Peng J Kitchen SM West RA Sigler R Eisenmann KM Alberts AS Myeloproliferative defects following targeting of the Drf1 gene encoding the mammalian diaphanous related formin mDia1 Cancer Res 2007 67 7565 7571 10.1158/0008-5472.CAN-07-1467 17699759

[B11] Zhu XL Liang L Ding YQ Overexpression of FMNL2 is closely related to metastasis of colorectal cancer Int J Colorectal Dis 2008 23 1041 1047 10.1007/s00384-008-0520-2 18665374

[B12] Karlsson R Pedersen ED Wang Z Brakebush C Rho GTPase function in tumorigenesis Biochim Biophys Acta 2009 1796 2 91 98 19327386 10.1016/j.bbcan.2009.03.003

[B13] Kitzing TM Wang Y Pertz O Copeland JW Grosse R Formin-like 2 drives amoeboid invasive cell motility downstream of RhoC Oncogene 2010 in press 20101212 10.1038/onc.2009.515

